# Assessing psychological health and reproductive function: Depression, anxiety, and stress in infertile men compared to controls: A case-control study

**DOI:** 10.18502/ijrm.v22i12.18068

**Published:** 2025-01-31

**Authors:** Seyedeh Narjes Roudbaraki, Maryam Ramezani, Bita Saifi, Mostafa Salimi, Massood Issapour Cheshani

**Affiliations:** ^1^Department of Psychology, Faculty of Medicine, Mashhad Medical Sciences, Islamic Azad University, Mashhad, Iran.; ^2^Student Research Committee, Faculty of Medicine, Mashhad Medical Sciences, Islamic Azad University, Mashhad, Iran.; ^3^Department of Basic Sciences, Faculty of Medicine, Mashhad Medical Sciences, Islamic Azad University, Mashhad, Iran.; ^4^Department of Urology, Faculty of Medicine, Mashhad Medical Sciences, Islamic Azad University, Mashhad, Iran.

**Keywords:** Male infertility, Stress, Anxiety, Depression.

## Abstract

**Background:**

With the increasing prevalence of infertility and its links to depression, anxiety, and stress, it is essential to compare these mental health levels between infertile men and a control group.

**Objective:**

This study aimed to compare depression, anxiety, and stress among infertile and fertile men. Also, assessing demographic factors affecting these challenges among both groups.

**Materials and Methods:**

This case-control study was conducted on 120 men at Milad Infertility Research Center, Mashhad, Iran from January 2023 to February 2023. Participants were divided into 2 groups: 60 infertile men and 60 healthy men who did not have fertility problems. Demographic information such as age, education, occupation, duration of the marriage, and duration of infertility was collected from their medical records, and they also completed the depression, anxiety, and stress scale 21 questionnaire through a telephonic interview. Finally, the findings were statistically analyzed.

**Results:**

Severe and very severe depression, anxiety, and stress were observed in 65%, 60%, and 43.4% of infertile men and 16.7%, 23.3%, and 11.7% of fertile men, respectively. Which was significantly more than the fertile group, and there was a significant relationship between depression (p ≤ 0.001), anxiety (p = 0.001), stress (p ≤ 0.001), and infertility. Also, a significant relationship was observed between the duration of infertility and depression (p = 0.031).

**Conclusion:**

Our study found infertile men had higher stress, anxiety, and depression than the control group. Limitations included phone-based data collection and the depression, anxiety, and stress scale 21 questionnaire's screening nature. Future studies should involve larger populations and consider economic status as a variable related to mental health.

## 1. Introduction

Parenting is an existential necessity, and the inability to achieve it can lead to severe psychological issues for couples, exacerbated by social pressures, isolation, and stigma. There are fewer studies on the psychosocial issues of male partners than on female partners (1). Both women and men suffer from the consequences of infertility, it can cause emotional distress, psychological challenges, and financial strain for couples. They may experience a range of emotions such as anger, guilt, sadness, anxiety, and a decline in self-confidence and self-esteem; and both may play a role in treatment (2). However, for many years, little attention has been paid to the psychosocial problems of infertile men (3). Several studies have shown that mental health factors, such as depression and anxiety, are related to the outcomes of assisted reproductive treatments and may contribute to the failure of the first course of these treatments (4, 5).

According to the definition of world health organization (WHO), infertility (a disease of the reproductive system) is the absence of pregnancy after having sex for 12 months or more if the person has not used any method of contraception. Infertility is one of the many medical problems in today's world. In such a way, it has increased by 90% since 1959, and now 15–10% of couples suffer from this problem (6). As there are about 80 million infertile people around the world, infertility is considered one of the most critical public health problems, by the WHO (7, 8). Infertility may be primary or secondary, in primary cases, the person has never experienced pregnancy, and in the secondary case, some people have previously had a history of fertility (9).

Infertility always brings various social, psychological, physical, and financial stress (10). According to a study conducted in Iran in 2020, the overall prevalence of infertility was reported as 7.8% (11). In the meantime, the prevalence of mental disorders in these people has been reported between 25% and 60% (12). Around 35–40% of infertility cases in couples are linked to men, with stress and anxiety also contributing to reduced fertility (13, 14). Stress and anxiety can reduce sperm count and mobility in men, as well as decrease sexual desire and frequency of intercourse. Conversely, infertility can lead to increased stress, creating a defective cycle (15).

Numerous studies indicate that both biomedical factors, like age and pregnancy history, as well as mental factors such as depression and anxiety, affect the outcomes of assisted reproductive treatments. These psychological states may even contribute to the failure of initial treatment attempts (16, 17).

Studying depression and anxiety in infertile men, along with the demographic factors that affect them, can help improve the identification and treatment of these issues. This understanding can lead to better outcomes in assisted reproductive treatments (18).

Considering the significance of fertility issues in Iran, the policies governing the country to increase the rate of fertility, alongside economic challenges, and the need to understand demographic factors influencing depression, anxiety, and stress, we decided to conduct the present study to compare depression, anxiety, and stress between infertile men and the control group.

## 2. Materials and Methods

### Study design

This case-control study was conducted on infertile men referred to the Milad Infertility Research Center, of Mashhad, Iran, and their fertile close relatives who had an age difference of ≤ 2 yr with them. The participants' information, such as age, education, occupation, duration of the marriage, and duration of infertility, was collected from their medical records in January 2023, and they were interviewed by phone in February 2023 to obtain informed consent and complete the questionnaire. 60 infertile men aged 20–40 yr who have at least 2 abnormal spermograms and their wives do not have a known infertility problem, referred to these infertility centers were included in the study.

Participants who had a history of neurological and psychiatric diseases, taking neuropsychiatric drugs in the last year, known physical problems, secondary infertility, or known causes of infertility in their women, were classified as exclusion criteria. This information was also checked according to the medical history and asked in the form of a checklist.

Then, the information form was completed for these cases, including age, education level, occupation, duration of marriage, and average duration of infertility.

In addition, the depression, anxiety, and stress scale 21 (DASS21)-questionnaire was completed for the participants to assess depression, anxiety, and stress scores. Also, 60 healthy men who do not have fertility problems have at least one child and are age-appropriate to the case group ( ≤ 2 yr age difference), were selected from the brothers or close relatives of the participants. They were included in the study as a control group, and the initial form and questionnaire were also completed for them.

### DASS21-questionnaire

This depression-anxiety-stress questionnaire measures depression-anxiety-stress scores and has 21 questions. The DASS-21 questionnaire includes 3 components; each sub-scale includes 7 questions, and the final score of each is obtained through the sum of the scores of the related questions. The scoring method is such that 0 (does not apply to me at all) for each question to 3 (incredibly applies to me) is considered. Since DASS-21 is the shortened form of the main scale (42 questions), the final score must be doubled for each subscale (19, 20).

They declared the validity of the DASS-21 questionnaire to be 0.77. Also, the reliability of the DASS-21 questionnaire and its components was obtained using Cronbach's alpha method for factors of psychological depression, anxiety, and stress, 0.89, 0.84, and 0.82, respectively (19). It should be mentioned that this questionnaire has been widely used in studies in Iran to assess depression, anxiety, and stress in various diseases and conditions (21, 22).

In a study titled “A study on the reliability and validity of the short form of the depression anxiety stress scale", the validity and reliability of this tool based on Cronbach's alpha coefficient for factors of psychological depression, anxiety, and stress were calculated 0.85, 0.75, and 0.87 respectively (23).

### Sample size

In another case-control study conducted in 2016, the average anxiety in 2 groups of infertile women and the control group was reported 3.2 ± 14.36 and 3.24 ± 3.69, respectively (24). The sample size was calculated considering the 95% confidence interval and 90% test power, and at least 60 men were in the case group and 60 in control group.


$$n=\frac{\left(S_{1}^{2}+S_{2}^{2}\right){\left(Z_{1-\frac{\alpha }{2}}+Z_{1-\beta }\right)}^{2}}{{\left({\bar{X}}_{2}-{\bar{X}}_{2}\right)}^{2}}=\frac{\left(3.2^{2}+3.24^{2}\right){\left(1.96+0.84\right)}^{2}}{{\left(14.36-3.69\right)}^{2}}=1.42$$

### Ethical Considerations

This research was approved by the Ethics Committee of Mashhad Medical Sciences, Islamic Azad University, Mashhad, Iran (Code: IR.IAU.MSHD.REC.1400.127). Informed consent was obtained from all applicants after explaining the conditions of the study in writing. They were also assured that all this information was confidential.

### Statistical Analysis

Mean and standard deviation were utilized to summarize quantitative variables. The nonparametric Mann-Whitney U test was employed to compare quantitative variables between 2 independent groups, while the Kruskal-Wallis test was used for comparison between 3 groups. Spearman's rank correlation was applied to assess the linear relationship between quantitative variables. The Chi-square test was conducted to analyze data on a nominal scale. Additionally, multiple linear regression analysis was performed to explore the relationships between variables. The study was carried out utilizing IBM-SPSS version 16, with a significance level of < 5%.

## 3. Results

The study compared depression, anxiety, and stress levels between infertile men and a control group. Variables like age, marriage duration, occupation, and education showed no significant differences between the groups. The case group had significantly higher average scores for depression, anxiety, and stress than the control group (p < 0.05) (Table I).

### Depression 

In the groups categorized by literacy -the illiterate or elementary school group, high school diploma group, and associate degree or higher group- the average depression score shows a statistically significant difference between the case and control groups (p < 0.05). In contrast, the middle school graduate group shows no significant difference in average depression scores between case and control groups (p > 0.05) (Table II).

For those married for ≤ 5 yr, average depression scores in the case and control groups show no significant difference. However, for marriages lasting 6–10 yr and > 10 yr, the case group reports significantly higher average depression scores. Additionally, a statistically significant difference in average depression scores is observed according to age in both groups (Table II).

While there is no statistically significant difference in depression scores based on the duration of infertility between the groups, Spearman's correlation analysis reveals a direct, significant, yet weak relationship between the duration of infertility and depression (Table II). Linear regression analysis showed no significant differences between the 2 study groups in age, marriage duration, occupation, and education levels (Table III).

### Anxiety 

No significant difference in anxiety scores was found for employees, but a significant difference was observed among self-employed men. In the groups of illiterate or elementary school group and those with a high school diploma, a statistically significant difference was observed in average anxiety scores between the case and control groups. Conversely, among middle school graduates with an associate degree or higher, anxiety scores did not differ significantly between groups (Table II).

There was no significant difference in scores for marriages lasting ≤ 5 yr between the case and control groups. However, significant differences were noted for those married for 6–10 yr and for those married for > 10 yr. Moreover, a statistically significant difference was observed in average anxiety scores based on age in both study groups (Table II).

The mean anxiety scores showed no significant differences between the 2 groups regarding infertility duration, and Spearman correlation analysis revealed no notable differences (Table II). The linear regression analysis showed no significant differences between the 1 study groups regarding age, marriage duration, occupation, and education level (Table III).

### Stress 

A significant difference in average stress scores based on job status was found in both case and control groups. In the groups categorized by education level -illiterate or elementary school group, high school diploma group, and associate degree or higher group- there exists a statistically significant difference in average stress scores between the case and control groups (p < 0.05). In middle school graduates, no significant difference was found in average stress scores between the case and control groups (Table II).

No statistically significant difference in average stress scores was found for couples married for ≤ 5 yr (p > 0.05). However, couples married for 6–10 yr showed a significant difference in average stress scores between the 2 groups. Additionally, no significant difference was found among men who have been married for more than 10 yr. A significant difference in average stress scores was observed by age in both groups (p < 0.05) (Table II).

The average stress scores showed no significant difference between groups regarding infertility duration, and Spearman's correlation analysis also indicated no significant differences (Table II). The linear regression analysis revealed no significant differences between study groups in age, marriage duration, job status, and education level (Table III).

In general, the variables of age, duration of marriage, level of education, and occupation did not show a statistically significant relationship with depression, anxiety, and stress scores between the 2 study groups. However, the linear regression analysis revealed significant differences between the case and control groups in situations of depression, anxiety, and stress scores (Table III). This indicates that male infertility is significantly linked to higher levels of depression, anxiety, and stress compared to a control group.

**Table 1:** Characteristics of study

**Variables**		**Case (n = 60)**	**Control (n = 60)**	**P-value**
Age (yr)*		34.72 ± 4.18	34.68 ± 4.11	0.86
Marriage duration (yr)*		7.84 ± 4.05	8.27 ± 4.27	0.30
**Infertility duration (yr)**		-	7.07 ± 0.25	-
Job**				
	**Self-employed**	85%	66.7%	0.19
	**Employee**	15%	33.3%	
Education**				
	**Illiterate or elementary school**	30%	25%	0.40
	**Middle school graduate**	30%	20%	
	**High school diploma**	23.3%	30%	
	**Associate degree or higher**	16.7%	25%	
Depression*		22.23 ± 8.43	16.43 ± 8.38	< 0.001
Anxiety*		16.40 ± 8.40	10.86 ± 7.44	< 0.001
Stress*		21.40 ± 8.64	14.43 ± 7.75	< 0.001
*Data presented as Mean ± SD, Mann-Whitney U test, **Data presented as percentages, Pearson Chi-square test				

**Table 2:** Comparison of depression, anxiety, and stress among groups

**Variables**		**Depression**			**Anxiety**			**Stress**		
		**Case**	**Control**	**P-value**	**Case**	**Control**	**P-value**	**Case**	**Control**	**P-value**
**Age (yr)**										
	**≤ 35**	31 (20.96 ± 7.94)	31 (13.74 ± 9.36)	< 0.001*	31 (16.64 ± 8.30)	31 (11.87 ± 8.42)	0.023*	31 (20.45 ± 8.68)	31 (14.58 ± 8.32)	0.007*
	**> 35**	29 (23.58 ± 8.87)	29 (13.72 ± 6.34)	< 0.001*	29 (16.20 ± 8.60)	29 (9.79 ± 6.19)	0.002*	29 (22.41 ± 8.64)	29 (14.27 ± 7.24)	0.001*
**Marriage duration (yr)**										
	**≤ 5**	19 (20.31 ± 8.49)	19 (15.05 ± 10.86)	0.072*	19 (15.26 ± 8.79)	19 (12.63 ± 9.73)	0.319*	19 (20.63 ± 8.59)	19 (15.78 ± 9.51)	0.098*
	**6-10**	32 (22.56 ± 8.08)	28 (13.21 ± 6.62)	< 0.001*	32 (16.43 ± 8.24)	28 (10.92 ± 5.56)	0.006*	32 (21.37 ± 8.81)	28 (13.28 ± 6.16)	< 0.001*
	**> 10**	9 (25.11 ± 9.54)	13 (12.92 ± 5.75)	0.004*	9 (18.88 ± 8.37)	13 (8.15 ± 6.90)	0.007*	9 (23.11 ± 8.89)	13 (14.92 ± 8.27)	0.051*
**Infertility duration (yr)**										
	**≤ 2**	8 (18.00 ± 8.48)	-	0.296** 0.031***	8 (12.25 ± 8.64)	-	0.405** 0.067***	8	-	0.604** 0.126***
								(19.00 ± 8.55)		
	**2-6**	17 (22.58 ± 6.73)			17 (16.70 ± 6.96)			17 (22.00 ± 7.24)		
	**> 6**	35 (23.02 ± 9.07)			35 (17.25 ± 8.87)			35 (21.65 ± 9.39)		
**Job**										
	**Self-employed**	51 (22.58 ± 8.33)	40 (15.05 ± 8.69)	< 0.001*	51 (16.66 ± 8.26)	40 (11.70 ± 7.32)	0.002*	51 (21.56 ± 8.60)	40 (15.85 ± 8.10)	0.002*
	**Employee**	9 (20.22 ± 9.24)	20 (11.10 ± 5.59)	0.02*	9 (15.11 ± 9.43)	20 (9.20 ± 7.57)	0.234*	9 (20.44 ± 9.36)	20 (11.60 ± 6.27)	0.013*
**Education**										
	**Illiterate or elementary school**	18 (24.90 ± 6.96)	15 (15.33 ± 10.01)	0.007*	18 (19.00 ± 9.26)	15 (11.86 ± 8.46)	0.015*	18 (23.22 ± 8.54)	15 (16.26 ± 9.55)	0.048*
	**Middle school graduate**	18 (20.66 ± 9.55)	12 (16.50 ± 8.57)	0.232*	18 (15.00 ± 6.90)	12 (13.66 ± 7.12)	0.415*	18 (19.88 ± 9.80)	12 (16.83 ± 6.84)	0.368*
	**High school diploma**	14 (22.57 ± 7.70)	18 (13.11 ± 6.96)	0.003*	14 (16.00 ± 7.35)	18 (9.33 ± 6.43)	0.013*	14 (21.85 ± 6.72)	18 (14.11 ± 7.27)	0.006*
	**Associate degree or higher**	10 (19.80 ± 9.54)	15 (10.66 ± 5.58)	0.016*	10 (15.00 ± 10.50)	15 (9.46 ± 7.65)	0.397*	10 (20.20 ± 9.58)	15 (11.06 ± 6.36)	0.014*

**Table 3:** Linear regression analysis

**Model**	**Depression**		**Anxiety**		**Stress**	
	**Beta**	**P-value**	**Beta**	**P-value**	**Beta**	**P-value**
**Groups (case-control)**	-0.429	< 0.001	-0.315	0.001	-0.363	< 0.001
**Age**	0.068	0.501	-0.037	0737	0.044	0.677
**Marriage duration**	-0.018	0.863	0.016	0.890	0.005	0.966
**Job**	-0.073	0.523	-0.059	0.634	-0.103	0.390
**Education**	-0.128	0. 267	-0.070	0.575	-0.070	0.562
Linear regression analysis to investigate the effect of the simultaneous relationship of variables on depression, anxiety, and stress						

## 4. Discussion 

In this study, we observed a statistically significant difference in the average scores for depression, anxiety, and stress between the case and control groups (p < 0.05), such that the average score in the case group was higher than the control group. A similar study consistent with our, showed that the degree of depression in infertile men was higher compared to healthy men, and severe anxiety was also estimated in men (9). In a study conducted in 2006, similar to our study, it was shown that the average score of depression in the infertile group and the control group was significantly different, and depression was higher in the infertile group compared to the fertile group (25).

One reason infertile men may score higher on depression, anxiety, and stress than fertile men are the pressure from family to have children. This pressure can affect both partners in an infertile couple. Many people dealing with infertility, fear an uncertain future after unsuccessful treatments along with the high costs associated with these procedures and their potential consequences, which can be distressing for many individuals experiencing infertility.

In a study conducted in Turkey, women had a significantly higher depression score than men participating in the study. However, unlike our study, no significant relationship between depression and male gender was found in the studied groups. In another study that aligns with our findings, a significant difference was observed in terms of anxiety between infertile couples and fertile couples, both in the male and female groups, while in contrast to our study, no significant difference was found regarding depression; this difference may be due to the use of different questionnaires (26).

In our study, the linear regression analysis conducted to examine the simultaneous relationships among variables revealed that job status did not have a significant impact on depression, anxiety, or stress in infertile men. Additionally, the level of education was found to have no significant correlation with these mental health outcomes. Furthermore, the duration of marriage also showed no significant relationship with depression, anxiety, or stress.

In some more studies related to ours, occupation and level of education were not related to depression and anxiety in infertile men (27, 28). In a study conducted in Iran in 2020, which is not consistent with our study, the level of stress and anxiety in infertile men had an inverse relationship with the level of education (18).

In several studies, which are similar to our study, the duration of marriage was not related to depression and anxiety in infertile men (25, 27, 28). In some studies, age was not related to depression and anxiety in infertile men, and our study confirms these studies (27–29).

When looking at the link between depression, anxiety, and stress scores and how long someone has been infertile, we found a meaningful connection just between infertility duration and depression (p < 0.05). This connection is steady and moderate, meaning that as the time of infertility gets longer, the depression scores tend to rise.

In one of the mentioned studies, no statistically significant difference was found between the duration of infertility and depression, anxiety, and stress among infertile men (28). In a study about depression in infertile and fertile couples in southern Iran, researchers found that people with a longer duration of infertility felt more anxiety than those with shorter infertility periods. Specifically, those who had been infertile for 1–3 yr experienced more anxiety compared to those who had been infertile for < 1 yr (25). Anxiety and depression were related to the duration of infertility as a result of a study, which was inconsistent with the case of anxiety and consistent with the case of depression in our study (30). In a Chinese study conducted in 2017, an infertility duration of more than 2 yr was associated with a higher risk of anxiety symptoms, which is inconsistent with our study (29).

## 5. Conclusion

In our study, there was a significant relationship between the duration of infertility in infertile men and depression, and the amount of stress, anxiety, and depression in infertile men was significantly higher than in the control group. The limitations of this research were the collection of information and questionnaires by phone, which led to the lack of accurate answers to some questions. Also, the DASS-21 questionnaire is used for screening, not a definitive diagnosis of depression, anxiety, and stress. In order to obtain more reliable results, it is suggested that future studies in this field be conducted in several public and private centers with a higher population. It is also recommended to evaluate the economic status of people as a variable and measure its relationship with depression, anxiety, and stress.

##  Data Availability

Data supporting the findings of this study are available upon reasonable request from the corresponding author.

##  Author Contributions

Concept and design: M. Issapour, S.N. Roudbaraki, B. Saifi. Data collection: M. Ramezani. Drafting of the manuscript: S.N. Roudbaraki, M. Salimi. Supervision: M. Issapour. Critical revision of the manuscript for important intellectual content: All authors.

##  Conflict of Interest

The authors declare that there is no conflict of interest.
